# An Artificial Intelligence Algorithm Integrated into the Clinical Workflow Can Ensure High Quality Acute Intracranial Hemorrhage CT Diagnostic.

**DOI:** 10.1007/s00062-024-01461-9

**Published:** 2024-09-26

**Authors:** K. Villringer, R. Sokiranski, R. Opfer, L. Spies, M. Hamann, A. Bormann, M. Brehmer, I. Galinovic, J. B. Fiebach

**Affiliations:** 1 Center for Stroke Research Berlin, Universitätsmedizin Berlin, Berlin, Germany; 2 Medizinische Versorgungszentren DRZ GmbH, Heidelberg, Germany; 3 jung diagnostics GmbH, Hamburg, Germany; 4 Klinik für Radiologie, Interventionsradiologie und Neuroradiologie, Klinikum Altenburger Land GmbH, Altenburg, Germany; 5 radprax MVZ Nordrhein GmbH, Wuppertal, Germany

**Keywords:** Artificial intelligence (AI), Cranial CT, Intracranial hemorrhage detection

## Abstract

**Purpose:**

Intracranial hemorrhage (ICH) is a life-threatening condition requiring rapid diagnostic and therapeutic action. This study evaluates whether Artificial intelligence (AI) can provide high-quality ICH diagnostics and turnaround times suitable for routine radiological practice.

**Methods:**

A convolutional neural network (CNN) was trained and validated to detect ICHs on DICOM images of cranial CT (CCT) scans, utilizing about 674,000 individually labeled slices. The CNN was then incorporated into a commercial AI engine and seamlessly integrated into three pilot centers in Germany. A real-world test-dataset was extracted and manually annotated by two experienced experts. The performance of the AI algorithm against the two raters was assessed and compared to the inter-rater agreement. The overall time ranging from data acquisition to the delivery of the AI results was analyzed.

**Results:**

Out of 6284 CCT examinations acquired in three different centers, 947 (15%) had ICH. Breakdowns of hemorrhage types included 8% intraparenchymal, 3% intraventricular, 6% subarachnoidal, 7% subdural, < 1% epidural hematomas. Comparing the AI’s performance on a subset of 255 patients with two expert raters, it achieved a sensitivity of 0.90, a specificity of 0.96, an accuracy of 0.96. The corresponding inter-rater agreement was 0.84, 0.98, and 0.96. The overall median processing times for the three centers were 9, 11, and 12 min, respectively.

**Conclusion:**

We showed that an AI algorithm for the automatic detection of ICHs can be seamlessly integrated into clinical workflows with minimal turnaround time. The accuracy was on par with radiology experts, making the system suitable for routine clinical use.

**Supplementary Information:**

The online version of this article (10.1007/s00062-024-01461-9) contains supplementary material, which is available to authorized users.

## Introduction

Intracranial hemorrhage (ICH) can be a serious complication of acute stroke with a risk varying from 2–7% [1] In ICH, on the other hand, premature mortality is as high as about 31 to 59% between 7 days and 1 year follow-up. ICH is also an increasingly more common side effect of anticoagulant therapy [2] besides intravenous thrombolysis (ivTPA) in the acute stroke setting. Moreover, the incidence of ICH increases with age [3] and thus plays an important role in a population getting increasingly older.

Cranial CT (CCT) without application of a contrast agent and, if necessary, a CT angiography is the standard of care in the acute stroke management. Prerequisite to administering ivTPA is the exclusion of ICH with a high degree of certainty and within the appointed time frame of 4.5 h after symptom onset.

Artificial Intelligence (AI) solutions are playing a more and more important role in radiological daily routine. They support radiologists in the detection and documentation of radiological findings as well as supporting in diagnosis.

So far, most AI solutions focused on the early detection of ischemia in stroke care [4]. Most studies showed that an additional AI evaluation minimizes the risk of overlooking an ICH in the acute setting as well [5–7]. Hence, a number of AI providers have established themselves whose solutions support the detection of both ischemia and ICH such as AIDOC (https://www.aidoc.com) and NICOLAB (https://www.nicolab.com) or scanner integrated AI solutions [6, 7] identifying hemorrhages in the routine clinical setting. The implementation of an AI solution can help providing radiological care at a high level even at night and during off-peak times. The general requirements are: (1) a high sensitivity, and thus few false-negative ICH findings and (2) the shortest possible response time (turnaround time), ideally less than 10 min, so that the AI result can also be taken into account in the acute setting and support therapy decision [8].

In this study, we will demonstrate how an AI algorithm provided by jung diagnostics GmbH (Hamburg, https://www.jung-diagnostis.de) was implemented as a cloud-based application in standard care and how its performance was tested under real-world conditions. As part of a pilot phase trial, the AI solution was tested in three district hospitals with acute care and a certified stroke unit in Germany.

The main question was, whether an AI analysis of CCT images that is optimally integrated into the workflow via a cloud service can ensure high quality ICH diagnostics in routine radiological care and with a turnaround time meeting the requirements of clinical routine also in the acute setting.

## Material and Methods

### Patients and Acquisition Protocols

The first pilot center (center-E) started on 04.03.2022. In the period from 04.03.2022 to 29.01.2024, all cranial CT scans (Siemens Emotion 16, axial, slice thickness 4 mm, matrix 512 × 512, pixel size 0.47 × 0.47 mm) were sent to the central server of jung diagnostics GmbH for evaluation. The same procedure was used for the period 28.12.2022 to 29.01.2024 for all cranial CT scans from center‑A (GE Medical Systems, Discovery CT750 HD, axial, slice thickness 5 mm, matrix 512 × 512, pixel size 0.43 × 0.43 mm) and in the period from 20.06.2023 to 30.11.2023 for all cranial CT scans (Siemens Somatom Definition; axial, slice thickness 5 mm; matrix 512 × 512, pixel 0.39 × 0.39 mm) from center‑R. The centers are small or medium regional or rural hospitals in Germany. Patients received a CCT either due to stroke or exclusion of intracranial hemorrhage after traumatic brain injury.

The cranial CT images were transferred anonymously to jung diagnostics GmbH under the conditions of the General Data Protection Regulation (GDPR) as part of the order processing. The ethics committee of the Hamburg Medical Association waived written consent for the retrospective use of the anonymized data (file number 2021-300047-WF).

### Real World Test-dataset to Determine the Performance of the AI Algorithm

The performance of the algorithm was assessed on data obtained consecutively over the period from 01.12.2022 to 28.02.2023 from one of the centers (center-E). The reason for the selection of the center was the availability of the lead radiologist in that center to serve as a manual rater with 25 years of neuroradiological experience (RS, rater1). In addition, two radiology specialists (IG and KV with 15 years of neuroradiological experience) shared the task of rating (rater2). The raters manually assessed each case independently if an ICH was present or not. Cases with missing DICOM (e.g. due to transfer problems) or insufficient image quality were sorted out by the raters. The image datasets were anonymized and independently assessed by the two raters. The evaluation followed a two-step approach. In the first step, both raters independently rated each case. In the second step, all cases with discrepancies between the ratings were re-evaluated by the raters (independently). The raters were then given the opportunity to either correct or confirm their initial assessments.

### AI Algorithm

**Fig. 1:**
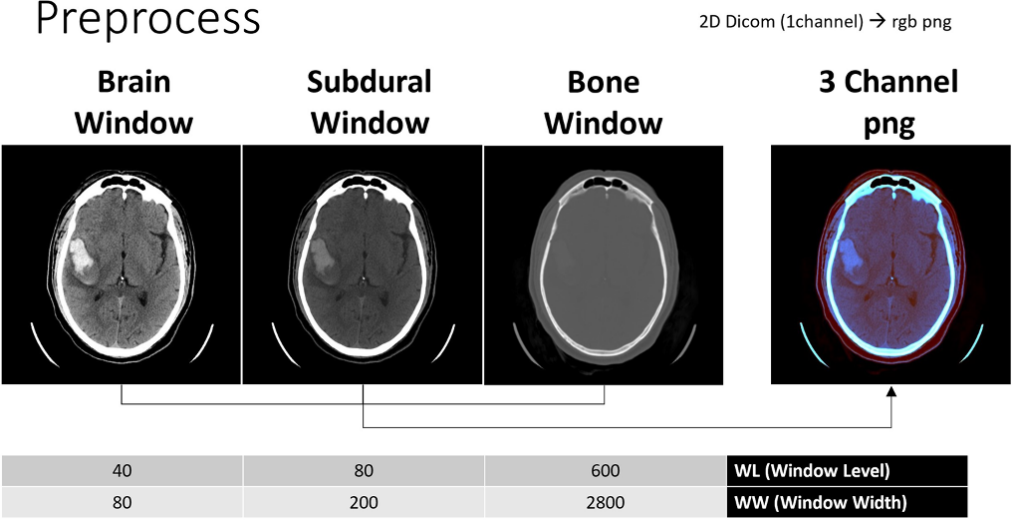
Illustration of the window level and window width for all three window types and the composition into an RGB image in PNG format

For slice-wise classification of ICH on the transferred DICOM slices, a convolution neural network (CNN) with an Efficient-Net-B3 architecture, as proposed by Tan, Le [9], was trained on 70% of approximately 674,000 individually labeled slices of the “RSNA Intracranial Hemorrhage Detection” dataset (https://www.kaggle.com/competitions/rsna-intracranial-hemorrhage-detection/data). The labels include a binary classification for the hemorrhage types intraparenchymal (IPH), intraventricular (IVH), subarachnoid (SAH), subdural (SDH) and epidural (EDH). In addition, an “Any” label was trained to indicate whether at least one of the listed hemorrhage types occurs on the respective slice. The loss function used was the binary cross-entropy loss between the binary labels and the probabilities *P*(x) ∈ [0.1] for the respective hemorrhage types after a final conv1 × 1&pooling and sigmoid activation function. For the input slice, a combination of three different window levels (WLs) were used in preprocessing, which were combined to form a 512 × 512 × 3 RGB channel, inspired by the winners of the RSNA2019 Intracranial Hemorrhage Detection Challenge (https://github.com/SeuTao/RSNA2019_Intracranial-Hemorrhage-Detection). The reduction of the gray values to Brain-Window (WL 30, Window-Width (WW) 80), Subdural-Window (WL 80, WW 200) and Bone-Window (WL 600, WW 2800) makes it possible to adjust the windows in advance to the one human examiners are used to (see Fig. 1).

The test and validation datasets each consisted of 15% of the DICOM slices of the RSNA dataset that were not used for training. The hyperparameters were adjusted on the validation dataset and threshold values were determined for each hemorrhage type based on the ROC curve of the test dataset in order to finally binarize the output of the network. The threshold value was selected so that the sum of sensitivity and specificity is maximized. A slice was considered positive for hemorrhage if both this and the “Any” label were above the respective fixed threshold value. Finally a complete case (all DICOM slices belonging to one patient) was judged to be positive if at least on one slice an ICH was detected.

The AI algorithm, approved as a medical device, was integrated into a commercial image analysis platform “Biometrica” (jung diagnostics GmbH, Hamburg, Germany).

### AI Post-processing

**Fig. 2:**
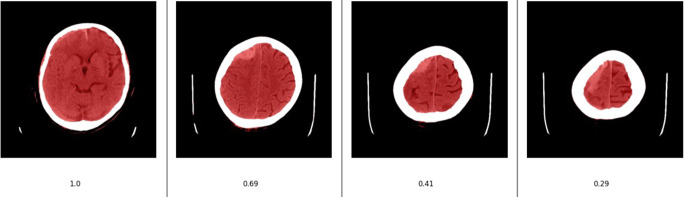
Example for the TIV segmentation (red) used to detect “upper-slices” in the postprocessing-step. The most left Image has the highest volume compared to all the other slices. Therefore the quotient of its volume and maximum volume is 1. The slice to the right only has 29% of that volume, which makes it fall below the threshold of 0.4 marking it as an “upper-slice”

Artifacts are very common near the skull, and streaks that mimic hemorrhage may appear due to beam hardening and scattering. Experienced radiologists consider slices with extensive bone incisions with caution. Therefore, hyperdensities near the upper skull were only considered hemorrhages if they appeared on two consecutive slices. This knowledge was integrated into the algorithm by developing the following rule set: A hemorrhage detected by the AI in the upper part of the skull is removed from this slice when no hemorrhage of the same type has been detected on a directly adjacent slice. To define the term “upper part” of the skull, a segmentation of the total intracranial volume (TIV), based on the threshold of the brain window, was performed. The area of the TIV was then determined for all slices and the maximum area was calculated from the total of all slices. A slice is called an “upper part ” if the area of the TIV is smaller than a certain percentage of the area of the slice with the most TIV. The threshold was set at 0.4 (see Fig. 2 for illustration). The analysis was repeated for each slice and each type of hemorrhage.

### Integration of AI into Standard Care

**Fig. 3:**
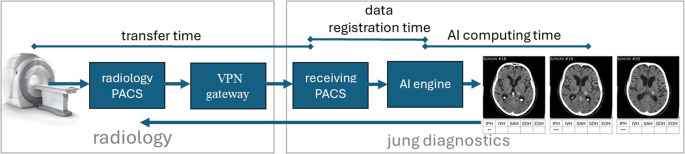
Schematic representation of the workflow and associated time intervals

The integration into the standard care workflow is schematically shown in Fig. 3. A pre-configured Virtual Private Network (VPN) gateway developed by jung diagnostics GmbH was integrated into the network of the respective hospital. As soon as the data from the modality reached the PACS, the CT-DICOM slices were sent to the DICOM node of the VPN gateway by means of automatic transfer via the DICOM send command. The VPN gateway de-identified and encrypted the data, which were then sent to the receiving PACS server (data center in Hamburg operated by jung diagnostic) via the VPN tunnel. The incoming data was then locally registered into a database and then automatically transferred to the allocated AI engine. The AI engine analyzed the data and created a PDF report. The PDF report was embedded into a DICOM object and sent back to the hospital’s PACS via the gateway in the same way. The documentation of findings consisted of displaying slices in which a hemorrhage was found, classifying the type of hemorrhage and assessing its probability, that is probable, very probable and almost certain a hemorrhage. An example report can be found in the supplemental material.

To minimize turnaround time, centers were asked to automate the transfer of data from the modality or internal PACS to the VPN gateway immediately upon completion of image acquisition.

### AI Versus Expert Raters On the Real-world Test Data

The result of the AI was compared with the final annotations of expert rater1 and rater2. Only the presence of an ICH was evaluated (binary result for each case). The categorization into the subtype as provided by the AI was ignored for this evaluation. The ratings of rater1 and rater2 were taken as ground truth (GT) and the AI results were compared against this GT. Each case was then categorized as true-positive (TP), false-positive (FP), true-negative (TN) and false-negative (FN). The sensitivity (SEN), the specificity (SPEC), the false positive ration (FPR), the accuracy (ACC), and the balanced accuracy (BalACC) was computed in the well-known standard way.

### Inter-rater Agreement On Real-world Test Data

The inter-rater variability was assessed by comparing the final ratings of rater1 against rater2 (and vice versa). The same parameters as described above were used (one rater was considered as the GT).

### Runtime Analysis and Computing Speed

The data was managed automatically using a workflow engine with an audit trail system proprietary to jung diagnostics GmbH. For all patients, a time stamp was automatically written to a database at each processing step, which could be evaluated for analyses. The following time periods were considered for the runtime analysis (see Fig. 3). Transfer time: the period from the acquisition of the data at the center to the delivery of the data to the receiving PACS server. Data registration time: time needed to register the incoming data and import the registered data into the local database. Since the receiving PACS was also used by many other radiology centers which were not related to this study a workflow engine was needed to orchestrate the data. The imaging study was then sent to the corresponding AI engine for image analysis. The computation time: time for AI analysis of the complete dataset and subsequent creation of the documentation.

The AI algorithm ran on a Linux server with 2 10-core CPUs (2.2 GHz, 13.75 MB) and 192 GB RAM. Graphics cards from NVIDIA (Quadro P5000 16 GB) were used.

## Results

After 7 days of training, the CNN achieved an area-under-the-curve (AUC) of 0.98 on the 101,137 (15%) slices of a test set of the RSNA dataset that was not included in the training. A slice-wise accuracy of 0.96, with a sensitivity of 0.80 and a specificity of 0.98, was achieved.

A total of 6284 native cranial CT examinations from routine care were successfully analyzed with the AI algorithm. Mean age was 72.6 years (y), the interquartile range was (IQR) [63.6 y, 85.1 y], and 53% were female. The analysis revealed 947 out of 6284 patients with ICH which corresponds to a proportion of 15%. The mean age of all positive cases was 74.9 y (IQR [66.9 y, 85.6 y], 48% female) The distribution of hemorrhages and the percentage per center can be found in Table 1.

**Table 1:** Distribution of ICHs across centers

Client	Number (n) of patients	ICH	IPH	IVH	SAH	SDH	EDH	Number of patients with >1 subtype
		*n* (% of all)	*n* (% of all; % of positive cases)					
Center‑A	2928	429 (15)	233 (8;54)	99 (3;23)	198 (7;46)	193 (7;45)	14 (< 1;3)	191 (7;45)
Center‑E	1914	321 (17)	132 (7;41)	53 (3;17)	105 (5;33)	183 (10;57)	15 (1;5)	108 (6;34)
Center‑R	1442	197 (14)	114 (8;58)	34 (2;17)	91 (6;46)	81 (6;41)	5 (< 1;3)	96 (7;49)
Total	6284	947 (15)	479 (8;51)	186 (3;20)	394 (6;42)	457 (7;48)	34 (< 1;4)	395 (6;42)

*ICH* intracranial hemorrhage, *IPH* intraparenchymal hemorrhage, *IVH* intraventricular hemorrhage, *SAH* subarachnoidal hemorrhage, *SDH* subdural hemorrhage, *EDH* epidural hemorrhage

### Performance of AI and Inter-rater Agreement On the Real-world Test Data

Of the 274 consecutive patients from center‑E considered for the performance analysis, 15 patients had fewer slices than the protocol allowed. These patients were excluded. For another 4 cases, there was no complete rating available because at least one of the raters skipped that case. These 19 cases were removed from the analysis and thus a total of 255 patients (mean age 75.2 y, IQR [66.9 y, 86.4 y], 49% female) were used for the final performance analysis (Table 2). The analysis showed that the AI (including the post-processing steps) had a mean (rater1 and rater 2) SEN of 0.90 and a mean SPEC of 0.04 with an overall BalACC of 0.93. The mean inter-rater agreement was 0.84 (SEN) with a mean SPEC of 0.96 and an overall BalACC of 0.91. The AI post-processing slightly improved the results. The number of FP findings was reduced from 14 to 8 for rater1 and from 14 to 9 for rater2. Without the post-processing step SEN was 0.91, SPEC 0.06 and BalACC 0.92.

**Table 2:** Performance of AI versus raters and inter-rater variability on 255 patients from the real-world test data

	TP	FN	FP	TN	SEN	FPR	SPEC	PPV	NPV	ACC	BalACC
rater1(GT) vs. AI	29	2	8	216	0.94	0.04	0.96	0.78	0.99	0.96	0.95
rater2(GT) vs. AI	28	5	9	213	0.85	0.04	0.96	0.76	0.98	0.95	0.90
*Mean AI vs. rater*	*28.5*	*3.5*	*8.5*	*214.5*	*0.90*	*0.04*	*0.96*	*0.77*	*0.98*	*0.96*	*0.93*
rater1(GT) vs. rater2	27	4	6	218	0.87	0.03	0.97	0.82	0.98	0.96	0.92
rater2(GT) vs. rater1	27	6	4	218	0.82	0.02	0.98	0.87	0.97	0.96	0.90
*Mean inter-rater*	*27*	*5*	*5*	*218*	*0.84*	*0.02*	*0.98*	*0.84*	*0.98*	*0.96*	*0.91*

*GT* ground truth, *TP* true positive, *FN* false negative, *FP* false positive, *TN* true negative, *SEN* sensitivity, *FPR* false positive rate, *SPEC* specificity, *PPV* positive predictive value, *NPV* negative predictive value, *ACC* accuracy, *BalACC* balanced accuracy

### Runtime Analysis

The results of the runtime analysis can be found in Table 3. The overall median time for the three centers was 644 s (sec) (for center-A), 729 sec (for center-E), and 552 sec (for center-R). The fastest part of the process was the AI computation time with a median of 18 sec for all three centers. The most time consuming part was the data transfer, partly because center‑E started the pilot trial with sending the imaging studies manually after completing the acquisition. Manual transfer requires attention by the staff and may be subject to delays and errors. The 90th percentile for data registration plus the AI computing time was less than 10 min for all three centers: 371 sec (352 sec +19 sec) for center‑A, 585 sec (529 sec +56 sec) for center‑E, and 421 sec (401 sec +20 sec) for center‑R.

**Table 3:** Runtime needed in seconds of the different process steps (see Fig. 3) for the three involved centers

Center	Process step	1st percentile	Median	90th percentile
Center‑A	Transfer time	471	535	637
Center‑E		161	593	5440
Center‑R		360	438	1052
Center‑A	Data registration time	42	91	352
Center‑E		53	118	529
Center‑R		45	96	401
Center‑A	AI computing time	16	18	19
Center‑E		16	18	56
Center‑R		16	18	20
Center‑A	Overall time	529	644	1008
Center‑E		230	729	6025
Center‑R		421	552	1473

## Discussion

We could demonstrate that a convolution neural network (CNN) with an Efficient-Net-B3 architecture [9] approach for the automatic detection of ICHs, seamlessly integrated into clinical workflow can detect ICH with high accuracy and minimal turnaround time.

The AI algorithm found a very similar fraction of patients with hemorrhage for all three centers (15% on average). A slightly higher numbers of hemorrhage as reported in the literature with 10.4% in Kiefer et al. [6] and 9.7% in Voter et al. [10]. A reason might be the higher age in our study. In another study with similar age (72 years) [11] a similar prevalence of 14.4% for all patients was identified in contrast to the studies mentioned above with a mean age of 68.7 years [6] and 61 years respectively [10]. The distribution of the different types of hemorrhages was quite similar across the different centers, however, differed from those in Kiefer et al. [6] and Rava et al. [7], probably due to the different nature of the clinical setting, that is an emergency department and stroke center in both.

There was a high inter-rater agreement (accuracy of 96%). This is consistent with inter-rater agreement reported in other studies [10, 12, 13]. There was no difference between the accuracy (0.96) of the AI and the inter-rater agreement (0.96). However, the AI was slightly more sensitive (i.e. fewer FN findings) than the raters (0.90 versus 0.84) to the expense of a lower specificity of the AI (0.96 versus 0.98, overall 8.5 FP versus 5 FP). The post-processing step removed some FP hyperdensities from the images, most likely caused by beam hardening and scatter effects. To better address these difficulties in future improvements the AI could be trained separately on slices near the upper part of the skull. This future work might remove the necessity to perform post-processing steps on the AI result.

**Fig. 4:**
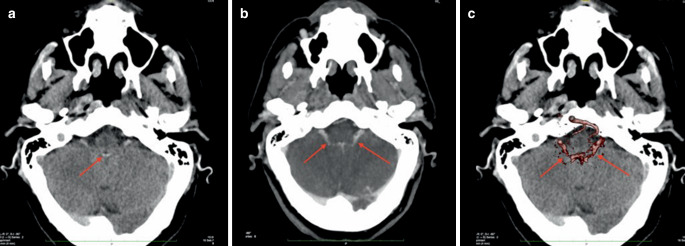
Example of a false-positive finding: the algorithm yielded a suspected intraventricular ICB. CT angiography shows that in this area of the suspected hemorrhage, choroid plexus vessels mimics a hemorrhage

For AI systems used as an alert or second read, false-negative findings (overlooked hemorrhage) are far more critical than false-positive findings (falsely detected hemorrhages) [14, 15]. Based on our results, the AI can play an important role, namely being a “watchful eye”, especially in stressful situations and in phases of lack of concentration [11, 16]. An example demonstrating the sensitivity of the presented AI is illustrated in Fig. 4. A hyperdensity of vascular structures or calcified choroid plexus was misjudged as hemorrhage by the AI.

The turnaround median time for the three centers was approximately 10 min. The AI computing time accounted for only a very small proportion of this turnaround time. The transfer time was by far the longest part in the process, followed by the data registration time. There was a great difference in the 90th percentile in transfer time and data registration time. This is partly due to the fact that the data transfer for Center‑E was not automated from the onset and because jung diagnostics currently uses one centralized data center for all image analysis services. Therefore, the transfer time and data registration time also depend on the total data traffic at jung diagnostics, which varies from day to day and time to time. Currently, jung diagnostics is setting-up a framework which allows for a flexible number of independent PACS servers, such that transfer time and data registration time will be independent from the overall data traffic in the future. This will further significantly reduce the turnaround time. Another solution to avoid long transfer times could be to set up servers on site in the hospital dedicated only for AI analysis. Then turnaround times of a few minutes could be achieved. Scanner installed AI solutions such as in Kiefer et al. [6] would be another approach to reduce turnaround time.

The AI solution from Aidoc (Aidoc, Tel Aviv, Israel) has become a standard in the AI-supported analysis of cranial CT images in recent years and is offered as a cloud-based solution (https://www.nice.org.uk/advice/mib207). In a recent paper, authors from a large Australian trauma clinic reported on their experiences and the performance of Aidoc under real-life conditions in the emergency department. They found a sensitivity of 82.1% (69.6–91.1%) and a specificity of 97.0% (95.6–98.0%) [8]. Various other studies reported a performance in the range of 88.7 to 96.2% for sensitivity and 92.3 to 99.0% for specificity [5, 7, 8]. In comparison to the aforementioned studies, our AI system achieved a sensitivity of 90% and a specificity of 96%, with a slightly better sensitivity but with a specificity which is within the performance range of Aidoc and thus corresponds to the state of the art and is close to that of the inter-rater variability with a sensitivity of 84% and specificity of 98%.

A limitation of this study is the underrepresentation of certain types of hemorrhage within the cohort used for performance analysis, which is due to the relatively small sample size (*n* = 255). Consequently, we were unable to differentiate into subtypes, as the number of positive findings was insufficient to achieve statistically significant results. It would be valuable to validate our findings using a larger dataset from additional centers, encompassing a wider variety of scanners. Despite this limitation, the algorithm was trained and evaluated on a diverse dataset of 674,000 images from various scanners. The algorithm demonstrated a 96% accuracy on the 15% test dataset, which is comparable to the measured accuracy of 96% on the 255 real-world test cases. This consistency suggests that the algorithm is robust across different scanner types.

The AI algorithm offers different levels of probability for the presence of hemorrhage along with its output thus reducing uncertainty of the radiologists decision. Contrary to Gibson et al. [17], which offers a colour-coded probability segmentation of intracranial hemorrhages, the AI algorithm transfers the probabilities into a report available to the radiologist as an aid in cases of uncertainty (supplemental material).

A major advantage of this study is the number of CCTs (*n* = 6284) examined in a real world scenario with high sensitivity and specificity contrary to those performed in tertiary referral hospitals with highly trained physicians [8], which makes the AI algorithm, as it is currently implemented, a suitable AI solution for all clinical settings.

The problem-free, serial evaluation of 6284 CT examinations in a multicenter setting including rural areas demonstrates the practical suitability of AI-supported analysis in routine standard care. A median total processing time, inclusive of transfer times of 9 min and 12 s was achieved for the fastest pilot center (center-R), where transfer times constitute the majority of the total time. Even the slowest of the three centers came close to a desired time of 10 min with a median of 12 min and 9 s. This means that one of the key requirements for AI has been met [8].

## Summary and Conclusion

This work shows the practical suitability of AI in acute diagnostics, which can achieve response times of a few minutes. Furthermore, the retrospective evaluation shows that the performance of AI is on average comparable to that of radiology experts.

The value of AI will be particularly high in situations where the aim is to achieve consistent diagnostic quality of care regardless of the radiologist’s level of experience. Particularly in challenging situations outside normal working hours, when qualified personnel are scarce or not immediately available, AI-supported analysis could provide greater certainty in the diagnosis of acute ICBs.

## Supplementary Information

Fig. 5 Example findings of the AI
