# Supplementary figures and images for: An Artificial Intelligence Algorithm Integrated into the Clinical Workflow Can Ensure High Quality Acute Intracranial Hemorrhage CT Diagnostic
Source: Clin Neuroradiol. 2024 Sep 26;35(1):115–22. doi: 10.1007/s00062-024-01461-9 (PMC11832613; doi:10.1007/s00062-024-01461-9)

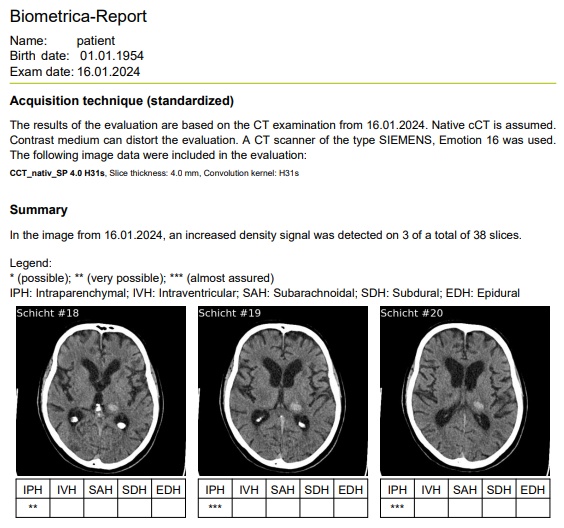


**Figure 5:** Example findings of the AI

Supplement: Supplementary file 1 — Fig. 5 Example findings of the AI [file 62_2024_1461_MOESM1_ESM.docx]
